# Healthcare-associated infections and antimicrobial resistance in Canadian acute care hospitals, 2018–2022

**DOI:** 10.14745/ccdr.v50i06a02

**Published:** 2024-06-28

**Authors:** Canadian Nosocomial Infection Surveillance Program

**Affiliations:** 1 Centre for Communicable Diseases and Infection Control, Public Health Agency of Canada, Ottawa, ON

**Keywords:** healthcare-associated infections, community-associated infections, antimicrobial resistance, surveillance, *Clostridioides difficile* infection, methicillin-resistant *Staphylococcus aureus*, vancomycin-resistant *Enterococcus*, carbapenemase-producing *Enterobacterales*, *Escherichia coli*, *Candida auris*, Canadian Nosocomial Infection Surveillance Program

## Abstract

**Background:**

Healthcare-associated infections (HAIs) and antimicrobial resistance (AMR) continue to contribute to excess morbidity and mortality among Canadians.

**Objective:**

This report describes epidemiologic and laboratory characteristics and trends of HAIs and AMR from 2018 to 2022 (*Candida auris*, 2012–2022) using surveillance and laboratory data submitted by hospitals to the Canadian Nosocomial Infection Surveillance Program (CNISP) and by provincial and territorial laboratories to the National Microbiology Laboratory.

**Methods:**

Data collected from 88 Canadian sentinel acute care hospitals between January 1, 2018, and December 31, 2022, for *Clostridioides difficile* infections (CDIs), carbapenemase-producing *Enterobacterales* (CPE) infections, methicillin-resistant *Staphylococcus aureus* (MRSA) bloodstream infections (BSIs) and vancomycin-resistant *Enterococcus* (VRE) BSIs. *Candida auris* (*C. auris*) surveillance was initiated in 2019 by CNISP and in 2017 (retrospectively to 2012) by the National Microbiology Laboratory. Trend analysis for case counts, rates, outcomes, molecular characterization and AMR profiles are presented.

**Results:**

From 2018 to 2022, decreased rates per 10,000 patient days were observed for CDIs (7% decrease; 5.42–5.02) and MRSA BSIs (2.9% decrease; 1.04–1.01). Infection rates for VRE BSIs increased by 5.9% (0.34–0.36). Infection rates for CPE remained low but increased by 133% (0.06–0.14). Forty-three *C. auris* isolates were identified in Canada from 2012 to 2022, with the majority in Western and Central Canada (98%).

**Conclusion:**

From 2018 to 2022, the incidence of MRSA BSIs and CDIs decreased and VRE BSI and CPE infections increased in the Canadian acute care hospitals participating in a national sentinel network (CNISP). Few *C. auris* isolates were identified from 2012 to 2022. Reporting standardized surveillance data to inform the application of infection prevention and control practices in acute care hospitals is critical to help decrease the burden of HAIs and AMR in Canada.

## Introduction

Healthcare-associated infections (HAI) represent one of the most common adverse events experienced by patients in acute care settings globally ((1)). In addition to increasing morbidity and mortality, they are associated with longer lengths of stay (LOS) in hospitals and higher costs of care. The prevalence of HAIs has been estimated to be at 3.2% in the United States (US), 6.5% in Europe and 9.9% in Australia, and is likely two-fold greater in developing countries ((1–4)). In Europe, the cumulative healthcare burden of six HAIs (urinary tract infection, pneumonia, surgical site infection, *Clostridioides difficile* infections [CDIs], bloodstream infections [BSIs], and neonatal sepsis) was greater than the burden of 32 other communicable diseases combined, including influenza and tuberculosis ((5)). In Canada, a point prevalence survey conducted in 2017 estimated that the prevalence of patients with at least one HAI was 7.9% ((6)). Importantly, a large proportion of HAIs are preventable and evidence from the US shows that advancements in care and infection prevention and control can decrease HAI rates over time ((2)).

Many of the microorganisms that cause HAIs have a propensity for antimicrobial resistance (AMR), and growing rates of resistance threaten to undermine efforts to reduce HAI rates ((5)). Infection with a resistant organism is associated with an 84.4% increased risk of death and in 2019, bacterial AMR was associated with approximately five million deaths globally ((7,8)). The global economic costs of AMR are also significant ((8)). Canadian data show that CDI is associated with a longer length of hospital stay, higher all-cause mortality and an average excess cost of $11,056 per patient ((9)).The rate of AMR is predicted to reach 40% by 2050. In this situation, it is forecasted that 13,700 Canadians could die each year from resistant infections, and the overall annual impact to Canada’s GDP would be $21 billion ((10)). Inappropriate antimicrobial use during the recent COVID-19 pandemic may have contributed towards an increase in AMR ((11)). Moreover, emerging resistant pathogens such as *Candida auris* (*C. auris*) have necessitated enhanced surveillance and changes to existing infection prevention and control protocols ((12)). Coordinated global public health action, surveillance, improved antibiotic stewardship, infection prevention and control and public awareness are crucial to identify patterns of antimicrobial resistance and prevent and control emerging infections.

In Canada, the Public Health Agency of Canada collects national data on various HAIs and AMR through the Canadian Nosocomial Infection Surveillance Program (CNISP). Established in 1994, CNISP is a collaboration between the Public Health Agency of Canada, the Association of Medical Microbiology and Infectious Disease Canada and sentinel hospitals from across Canada. The goal of CNISP is to facilitate and inform the prevention, control and reduction of HAIs and antimicrobial resistant organisms in Canadian acute care hospitals through active surveillance and reporting.

In line with the World Health Organization’s core components of infection prevention and control ((13)), CNISP performs consistent, standardized surveillance to reliably estimate HAI burden, establish benchmark rates for national and international comparison, identify potential risk factors and assess and inform specific interventions to improve patient health outcomes. Data provided by CNISP directly support the collaborative goals outlined in the *Pan-Canadian Action Plan on Antimicrobial Resistance* ((14)).

In this report, we describe the most recent HAI and AMR surveillance data collected from CNISP participating hospitals between 2018 and 2022. Further, we provide a summary of *C. auris* isolates identified from 2012 to 2022 to describe the epidemiology of this pathogen in Canada.

## Methods

### Design

The CNISP conducts prospective, sentinel surveillance for HAIs (including antimicrobial resistant organisms) ((15)).

### Case definitions

Standardized case definitions for healthcare-associated (HA) and community-associated (CA) infections were used. Refer to **Appendix A** for full-case definitions.

### Data sources

Between January 1, 2018, and December 31, 2022, participating hospitals submitted epidemiologic data and isolates for cases meeting the respective case definitions for CDI, methicillin-resistant *Staphylococcus aureus* (MRSA) BSIs, vancomycin-resistant *Enterococcus* (VRE) BSIs and carbapenemase-producing *Enterobacterales* (CPE) infections. Eligible *C. auris* isolates (infections or colonizations) were identified by provincial and territorial laboratories and participating hospital laboratories between January 1, 2012, and December 31, 2022, while CNISP surveillance for clinical characteristics of *C. auris* began on January 1, 2019. In 2022, 88 hospitals in 10 provinces and one territory participated in HAI surveillance and are further described in **Table 1** and **Appendix B**, **Supplemental Figure S1**. Hospital participation varied by surveillance project and year. In 2022, patient admissions captured in CNISP HAI surveillance were distributed across hospitals categorized as either small (1–200 beds, n=39 sites, 44%), medium (201–499 beds, n=34 sites, 39%) or large (500 or more beds, n=15 sites, 17%) (Table 1).

**Table 1:** Summary of hospitals participating in the Canadian Nosocomial Infection Surveillance Program, by region, 2022

Details of participating hospitals	Western^a^	Central^b^	Eastern^c^	Northern^d^	Total
Total number of hospitals	29	32	26	1	88
**Hospital type**					
Adult^e^	12	21	16	0	49
Mixed	13	7	9	1	30
Paediatric	4	4	1	0	9
**Hospital size**					
Small (1–200 beds)	11	7	20	1	39
Medium (201–499 beds)	10	19	5	0	34
Large (500 or more beds)	8	6	1	0	15
**Admissions and discharge**					
Total number of beds	10,031	11,772	3,258	25	25,086
Total number of admissions	444,247	518,799	107,324	2,313	1,072,683
Total number of patient days	3,653,051	4,048,979	993,560	7,046	8,702,636

^a^ Western refers to British Columbia, Alberta, Saskatchewan and Manitoba

^b^ Central refers to Ontario and Québec

^c^ Eastern refers to Nova Scotia, New Brunswick, Prince Edward Island and Newfoundland and Labrador

^d^ Northern refers to Yukon, Northwest Territories and Nunavut

^e^ Eleven hospitals classified as “adult” had a neonatal intensive care unit

Epidemiologic (demographic, clinical and outcomes) and denominator data (patient days and patient admissions) were collected and submitted by participating hospitals through the Canadian Network for Public Health Intelligence, a secure online data platform.

Reviews of standardized protocols and case definitions are conducted annually by established infectious disease expert working groups; training for data submission is provided to participating CNISP hospital staff as required. Data quality for surveillance projects is periodically evaluated; additional details on the methodology have been published previously ((16,17)).

### Laboratory data

All patient-linked laboratory isolates (stool samples for CDI cases) were sent to the Public Health Agency of Canada’s National Microbiology Laboratory for molecular characterization and antimicrobial susceptibility testing. Isolates for MRSA BSIs, VRE BSIs, CPE infections, *C. auris* (2019–2022) infections and paediatric CDIs were submitted year-round. Adult CDI isolates were submitted annually during a targeted two-month period (March 1 to April 30).

### Statistical analysis

Rates of HAI were calculated by dividing the total number of cases identified in patients admitted to CNISP participating hospitals by the total number of patient admissions (multiplied by 1,000) or patient days (multiplied by 10,000). The HAI rates are reported nationally and by region as shown in Table 1. Sites that were unable to provide case data were excluded from rate calculations and missing denominator data were estimated using their previous years reported data, where applicable. Missing epidemiological and molecular data were excluded from analysis. The Mann-Kendall test was used to test trends. Significance testing was two-tailed and differences were considered significant at *p*≤0.05.

Where available, attributable and all-cause mortality were reported for HAIs. Attributable mortality rate was defined as the number of deaths per 100 HAI cases where the HAI was the direct cause of death or contributed to death within 30 days of positive culture or histopathology specimen, as determined by physician review. All-cause mortality rate was defined as the number of deaths per 100 HAI cases 30 days following positive culture.

## Results

### *Clostridioides difficile* infection

Between 2018 and 2022, overall CDI rates decreased by 7% (5.42 to 5.02 infections per 10,000 patient days); however, this trend was not significant (*p*=0.327) (**Table 2**). Stratified by source of infection, the incidence of HA-CDI showed a non-significant decrease of 7.3% from 3.95 to 3.66 infections per 10,000 patient days (*p*=0.327) (Appendix B, **Table S1.1**). Community-associated-CDI rates remained stable when comparing 2018 to 2022 rates per 1,000 patient admissions (Appendix B, Table S1.1).

**Table 2:** *Clostridioides difficile* infection data, Canada, 2018–2022^a^

*C. difficile* infection data	Year									
	2018		2019		2020		2021		2022	
**Number of infections and incidence rates**										
Number of *C. difficile* infection cases	3,850		3,600		3,654		3,643		3,846	
Rate per 1,000 patient admissions	4.19		3.73		4.14		3.99		4.18	
Rate per 10,000 patient days	5.42		4.90		5.35		5.06		5.02	
Number of reporting hospitals	68		73		82		80		72	
Attributable mortality rate per 100 cases (%)^b^	1.3		2.3		2.7		2.4		1.1	
**Antimicrobial resistance^c^**	**n**	**%**	**n**	**%**	**n**	**%**	**n**	**%**	**n**	**%**
Clindamycin	307	48.7	221	38.9	62	17.1	67	12.4	94	23.6
Moxifloxacin	70	11.1	66	11.6	24	6.6	49	9	29	7.3
Rifampin	10	1.6	6	1.1	3	0.8	9	1.7	4	1.0
Metronidazole	1	0.2	0	0	0	0	0	0	0	0
Total number of isolates tested^d^	630	N/A	568	N/A	363	N/A	542	N/A	399	N/A

Abbreviations: *C. difficile, Clostridioides difficile*; N/A, not applicable

^a^ All *C. difficile* isolates from 2017 to 2021 submitted to the National Microbiology Laboratory were susceptible to tigecycline and vancomycin

^b^ Deaths where *C. difficile* infection was the direct cause of death or contributed to death 30 days after the date of the first positive lab specimen or positive histopathology specimen. Mortality data are collected during the two-month period (March and April of each year) for adults (aged 18 years and older) and year-round for children (aged one year to younger than 18 years old). Among paediatric patients, there was no death attributable to healthcare-associated *C. difficile* infection

^c^ *C. difficile* infection isolates are collected for resistance testing during the two-month period (March and April of each year) for adults (aged 18 years and older) and year-round for children (aged one year to younger than 18 years old) from admitted patients only

^d^ Total number reflects the number of isolates tested for each of the antibiotics listed above

Regionally, HA-CDI rates have decreased across all regions except the East where rates have increased by 11.7% (*p*=0.33), but this result is not significant. For CA-CDI, rates remain highest overall in the Central region from 2018 and 2022 (range: 1.39–1.66), followed by West and East. Overall CDI attributable mortality remained low and fluctuated (range: 1.1–2.7 deaths per 100 cases) from 2018 to 2022 (*p*=1.00) (Appendix B, Table S1.1).

From 2018 to 2022, 35.9% (n=897/2,501) of CDI isolates were resistant to one or more tested antimicrobials. The proportion of *C. difficile* isolates resistant to moxifloxacin decreased by 3.8% between 2018 (11.1%, n=70/630) and 2022 (7.3%, n=29/399) (Table 2). Since 2018, moxifloxacin resistance decreased non-significantly among HA-CDI isolates (4.7%, *p*=0.142) while a smaller non-significant decrease was observed among CA-CDI (1.0%, *p*=0.142) (Appendix B, **Table S1.2**). All tested *C. difficile* isolates were susceptible to vancomycin and tigecycline. From 2018 to 2022, the prevalence of ribotype RT027 associated with NAP1 decreased by 4.8% from 8.4% to 3.6% and 1.2% from 3.2% to 2.0%, respectively (Appendix B, **Table S1.3**).

### Methicillin-resistant *Staphylococcus aureus* bloodstream infections

Between 2018 and 2022, overall MRSA BSI rates decreased by 2.9% (1.04 to 1.01 infections per 10,000 patient days), with a peak rate observed in 2020 (1.16 infections per 10,000 patient days) (**Table 3**). Stratified by case type, a continued steady increase of 12% (0.5 to 0.56 infections per 10,000 patient days, *p*=0.05) was observed from 2018 to 2022 in CA-MRSA BSI rates. The HA-MRSA BSI rates remained stable over time (range: 0.42–0.50 infections per 10,000 patient days) (Appendix B, **Table S2.1**).

**Table 3:** Methicillin-resistant *Staphylococcus aureus* bloodstream infections data, Canada, 2018–2022

MRSA BSI data	Year									
	2018		2019		2020		2021		2022	
**Number of infections and incidence rates**										
Number of MRSA BSIs	764		881		868		874		820	
Rate per 1,000 patient admissions	0.77		0.85		0.88		0.86		0.83	
Rate per 10,000 patient days	1.04		1.14		1.16		1.13		1.01	
Number of reporting hospitals	62		69		81		80		78	
**All-cause mortality rate^a^**										
Number of deaths	144		144		152		165		162	
All-cause mortality rate per 100 cases	18.8		16.3		17.5		18.9		19.8	
**Antimicrobial resistance^b^**	**n**	**%**	**n**	**%**	**n**	**%**	**n**	**%**	**n**	**%**
Ciprofloxacin	502	71.8	561	70.5	460	65.6	488	65.9	389	65.4
Clindamycin	287	41.1	297	37.3	234	33.4	221	29.8	147	24.7
Erythromycin	527	75.4	603	75.8	507	72.3	508	68.6	403	67.7
Gentamicin	28	4.0	35	4.4	22	3.1	36	4.9	20	3.4
Rifampin	6	0.9	7	0.9	6	0.9	9	1.2	5	0.8
Trimethoprim/sulfamethoxazole	12	1.7	15	1.9	16	2.3	32	4.3	35	5.9
Tetracycline	49	7.0	62	7.8	46	6.6	64	8.6	49	8.2
Tigecycline	0	0	0	0	1	0.1	6	0.8	5	0.8
Total number of isolates tested^c,d^	699	N/A	796	N/A	701	N/A	741	N/A	595	N/A

Abbreviations: MRSA BSI, methicillin-resistant *Staphylococcus aureus* bloodstream infection; N/A, not applicable

^a^ Based on the number of cases with associated 30-day outcome data

^b^ All MRSA isolates from 2018 to 2022 submitted to the National Microbiology Laboratory were susceptible to linezolid, nitrofurantoin and vancomycin

^c^ In some years, the number of isolates tested for resistance varied by antibiotic

^d^ Total number reflects the number of isolates tested for each of the antibiotics listed above

In 2022, HA and CA-MRSA BSI rates were highest in Western Canada (0.48 and 0.71 infections per 10,000 patient days, respectively) (Appendix B, Table S2.1). Among hospital types, HA and CA-MRSA BSI rates have generally remained highest among adult and mixed hospitals. Stratified by hospital size, rates of HA-MRSA BSI were highest among medium (201–499 beds) and large size hospitals (500 or more beds) while CA-MRSA BSI rates have been highest in medium size hospitals since 2019 (Appendix B, Table S2.1). All-cause mortality remained relatively stable from 2018 to 2022 (range: 16.3%–19.8%) (Table 3). In 2022, 30-day all-cause mortality was higher among those with HA-MRSA (23.6%) compared to those with CA-MRSA (17.5%) (*p*=0.034).

Clindamycin resistance among MRSA isolates decreased significantly by 16.4% between 2018 and 2022 (2018: 41.1%, n=287/699; 2022: 24.7%, n=147/595) (*p*=0.0143) (Table 3). Since 2018, the proportion of MRSA isolates with erythromycin and ciprofloxacin resistance decreased, yet remained high (67.7%, n=403/595 and 65.4%, n=389/595 in 2022, respectively) in relation to other antibiotics tested. All submitted MRSA BSI isolates from 2018 to 2022 were susceptible to linezolid, nitrofurantoin and vancomycin.

Comparing HA-MRSA isolates to CA-MRSA isolates, clindamycin resistance was consistently higher among HA-MRSA isolates each year from 2018 (50.0%, n=166/332 vs. 33.0%, n=110/333) to 2022 (28.8%, n=68/236 vs. 21.9%, n=73/334) (Appendix B, **Table S2.2**). There were no other notable differences in antibiotic resistance patterns by MRSA BSI case type.

Between 2018 and 2022, the proportion of spa types identified as t002 (CMRSA2) and most commonly associated with HA-MRSA continued to decrease from 25.3% of all HA-MRSA isolates in 2018 to 6.4% in 2022. The proportion of spa types identified as t008 (CMRSA10) and most commonly associated with CA-MRSA continued to increase and account for the largest proportion of CA-MRSA isolates from 45.0% in 2018 to 49.1% in 2022 (Appendix B, **Table S2.3**).

### Vancomycin-resistant *Enterococcus* bloodstream infections

From 2018 to 2022, VRE BSI rates increased by 5.9%, from 0.34 to 0.36 infections per 10,000 patient days (**Table 4**). Regionally, VRE BSI rates were highest in Western and Central Canada (0.52 and 0.31 infections per 10,000 patient days in 2022, respectively) with few VRE BSIs reported in Eastern Canada (range: 0–0.02 infections per 10,000 patient days) (Appendix B, **Table S3.1**). Stratified by hospital type, VRE BSI rates remained highest in adult hospitals from 2018 to 2022 (range: 0.38–0.47 infections per 10,000 patient days). From 2018 to 2022, VRE BSI rates in paediatric hospitals were low (range: 0–0.25 infections per 10,000 patient days). In 2022, VRE BSI rates were 0.47 infections per 10,000 patient days in large hospitals (500 or more beds), 0.32 infections per 10,000 patient days in medium hospitals (201–499 beds) and 0.17 infections per 10,000 patient days in small hospitals (1–200 beds).

**Table 4:** Vancomycin-resistant *Enterococcus faecium* bloodstream infections data, 2018–2022

VRE BSI data	Year									
	2018		2019		2020		2021		2022	
**Vancomycin-resistant *Enterococcus* bloodstream infections data**										
Number of VRE BSIs	242		241		224		251		302	
Rate per 1,000 patient admissions	0.25		0.23		0.23		0.25		0.29	
Rate per 10,000 patient days	0.34		0.30		0.30		0.32		0.36	
Number of reporting hospitals	61		70		81		80		80	
**Antimicrobial resistance of *Enterococcus faecium* isolates**	**n**	**%**	**n**	**%**	**n**	**%**	**n**	**%**	**n**	**%**
Ampicillin	181	98.9	173	100	130	97.0	164	98.8	193	98.0
Chloramphenicol	5	2.7	30	17.3	28	20.9	52	31.3	32	16.2
Ciprofloxacin	183	100	173	100	131	97.8	164	98.8	196	99.5
Daptomycin^a^	11	6.0	7	4.0	4	3.0	4	2.4	4	2.0
Erythromycin	175	95.6	166	96.0	127	94.8	157	94.6	192	97.5
High-level gentamicin	79	43.2	57	32.9	35	26.1	32	19.3	37	18.8
Levofloxacin	181	98.9	173	100	130	97.0	164	98.8	195	99.0
Linezolid	2	1.1	3	1.7	1	0.7	3	1.8	6	3.0
Nitrofurantoin	54	29.5	66	38.2	54	40.3	129	77.7	138	70.1
Penicillin	181	98.9	173	100	131	97.8	164	98.8	194	98.5
Quinupristin/dalfopristin	21	11.5	18	10.4	8	6.0	8	4.8	15	7.6
Rifampicin	163	89.1	160	92.5	114	85.1	153	92.2	182	92.4
High-level streptomycin	62	33.9	42	24.3	29	21.6	48	28.9	48	24.4
Tetracycline	110	60.1	119	68.8	88	65.7	132	79.5	175	88.8
Tigecycline	1	0.5	0	0.0	0	0.0	0	0.0	1	0.5
Vancomycin	178	97.3	170	98.3	129	96.3	161	97.0	196	99.5
Total number of isolates tested^b^	183	N/A	173	N/A	134	N/A	166	N/A	197	N/A

Abbreviations: N/A, not applicable; VRE BSI, vancomycin-resistant *Enterococcus* bloodstream infection

^a^ Clinical and Laboratory Standards Institute (CLSI) resistance breakpoints came into effect in 2019 and was applied to all years

^b^ Total number reflects the number of isolates tested for each of the antibiotics listed above

Note: Aggregate mortality data reported in-text due to fluctuations in the small numbers of VRE BSI deaths reported each year

Vancomycin-resistant *Enterococcus* BSIs were predominantly HA, as 90.1% (n=1,135/1,260) of VRE BSIs reported from 2018 to 2022 were acquired in a healthcare facility. All-cause mortality remained high (34%) from 2018 to 2022. The incidence rates by region, hospital type and hospital size are presented in Appendix B, **Table S3.2**.

Between 2018 to 2022, high-level gentamicin resistance among VRE BSI isolates (*Enterococcus faecium*) decreased from 43.2% to 18.8% (*p*=0.01) (Table 4). Daptomycin non-susceptibility, first identified in 2016, decreased from 6.0% (n=11 isolates) in 2018 to 2.0% (n=4 isolates) in 2022 (*p*=0.0143). Since 2018, the majority (99.3%) of VRE BSI isolates were identified as *E. faecium*; however, three *Enterococcus faecalis* (*E. faecalis*) were identified in 2018 and one in each of 2020, 2021 and 2022 (Appendix B, **Table S3.3**). Among *E. faecium* isolates, the proportion identified as sequence type (ST)1478 was highest in 2018 (37.2%, n=67/180) and decreased to 8.7% (n=17/196) in 2022 (*p*=0.05) (Appendix B, **Table S3.4**). Furthermore, the proportion of ST17 isolates significantly increased from 2018 (5.0% n=9/180) to 2022 (46.9%, n=92/196) (*p*=0.05) (Appendix B, Table S3.4).

### Carbapenemase-producing *Enterobacterales*

From 2018 to 2022, CPE infection rates have remained low, although there has been a non-significant increase of 133% in the rates over this period (0.06 to 0.14 infections per 10,000 patient days, *p*=0.07) (**Table 5**).

**Table 5:** Carbapenemase-producing *Enterobacterales* data, Canada, 2018–2022^a,b^

CPE data	Year									
	2018		2019		2020		2021		2022	
**Number of infections and incidence rates**										
Number of CPE infections	36		54		41		73		107	
Infection rate per 1,000 patient admissions	0.05		0.06		0.05		0.08		0.11	
Infection rate per 10,000 patient days	0.06		0.08		0.06		0.11		0.14	
Number of reporting hospitals	50		60		72		73		77	
**Carbapenemases identified**	**n**	**%**	**n**	**%**	**n**	**%**	**n**	**%**	**n**	**%**
KPC	115	52.3	131	45.3	98	40.0	142	47.4	143	51.4
NDM	55	25.0	104	32.1	80	32.7	80	26.3	60	21.6
OXA-48	30	13.6	46	14.1	48	19.6	47	15.5	64	23.0
SME^c^	4	1.8	1	0.3	2	0.8	1	0.3	0	0.0
NDM/OXA-48	6	2.7	16	4.9	9	3.7	12	3.9	5	1.8
GES	1	0.5	1	0.3	0	0.0	1	0.3	0	0.0
IMP	3	1.4	1	0.3	1	0.4	1	0.3	2	0.7
NMC	2	0.9	4	1.2	7	2.9	15	4.9	2	0.7
VIM	2	0.9	3	0.9	0	0.0	1	0.3	2	0.7
Other	2	0.9	2	0.6	0	0.0	2	0.7	0	0.0
Total number of isolates tested^d^	220	N/A	327	N/A	245	N/A	304	N/A	278	N/A

Abbreviations: CPE, carbapenemase-producing *Enterobacterales*; GES, Guiana extended-spectrum β-lactamase; IMP, active-on-imipenem; KPC, *Klebsiella pneumoniae* carbapenemase; NDM, New Delhi metallo-β-lactamase; NMC, not metalloenzyme carbapenemase; N/A, not applicable; OXA-48, Oxacillinase-48; SME, *Serratia marcescens* enzymes; VIM, Verona integron-encoded metallo-β-lactamase

^a^ Includes data for all CPE isolates submitted

^b^ *Enterobacter cloacae* complex includes *Enterobacter cloacae* and other *Enterobacter* spp.

^c^ Only found in *Serratia marcescens*

^d^ Some isolates contain multiple carbapenemases therefore the total number of isolates tested and the number of carbapenemases indicated may not match. *Acinetobacter baumanii* were not included in this table

Note: Aggregate mortality data reported in-text due to fluctuations in the small numbers of CPE deaths reported each year

From 2018 to 2022, the majority of CPE infections (98.0%) were identified in Central (52.1%, n=162/311) and Western Canada (46.0%, n=143/311) while few infections were identified in the East (1.9%, n=6/311) (Appendix B, **Table S4.1**). From 2018 to 2022, large hospitals (500 or more beds) generally reported the highest rates of CPE infections (0.07–0.17 infections per 10,000 patient days). Thirty days all-cause mortality was 16.3% (n=46/282). During this period, 22.8% (n=68/298) of CPE-infected patients reported travel outside of Canada and of those, 67.6% (n=46/68) received medical care while abroad.

The predominant carbapenemases identified in Canada were *Klebsiella pneumoniae* (*K. pneumoniae*) carbapenemase, New Delhi metallo-β-lactamase and Oxacillinase-48 (OXA-48), accounting for 86.0% to 96.0% of identified carbapenemases from 2018 to 2022. Among submitted isolates, *Escherichia coli* remains the most commonly identified carbapenemase-producing pathogen from 2018 to 2022 (range: 23.0%–34.1%) (Appendix B, **Table S4.2**). From 2018 to 2022, carbapenemase-producing pathogens identified as *K. pneumoniae* decreased by 7.0% while *Citrobacter freundii* increased by 6.2%. (Appendix B, Table S4.2). Among the predominant cabapenemases, from 2019 to 2022, the prevalence of meropenem resistance among *K. pneumoniae* carbapenemase isolates decreased by 13.3%. Among New Delhi metallo-β-lactamase isolates, the prevalence of aztreonam resistance decreased by 13%, while amikacin resistance increased by 10.8%. Among OXA-48 isolates, the largest decreases in resistance were seen in ceftriaxone, tobramycin and trimethorpim/sulfamethoxazole (26.6%, 22.5% and 22.3%, respectively) (Appendix B, **Table S4.3 to S4.5**).

### Candida auris

A total of 43 isolates (colonizations and infections) have been reported to National Microbiology Laboratory from 2012 to 2022, of which eight had detailed CNISP patient questionnaires completed. Twenty-one cases were from Western Canada, 21 cases were from Central Canada and one case was reported from Eastern Canada. Approximately, one third of isolates were resistant to amphotericin B (34.9%, n=15/43) and two thirds were resistant to fluconazole (67.4%, n=29/43). One third of isolates were multidrug-resistant (resistant to two classes of antifungals) (34.9%, n=15/43). Of the 13 patients with travel information, four reported no travel (31%) while nine reported international travel (69%). Of the nine patients with reported history of travel, eight had received health care abroad (89%). Of the eight patients who received travel abroad, six had known carbapenemase-producing organism status and three tested positive.

## Discussion

Canadian Nosocomial Infection Surveillance Program surveillance data have shown that between 2018 and 2022, infection rates in Canada have decreased for CDI and MRSA BSI (7.0% and 2.9%, respectively). Rates have increased for VRE BSI and CPE infection (5.9% and 133%, respectively). A total of 43 *C. auris* isolates were identified from 2012 to 2022.

Declining CDI rate trends observed in the CNISP network follow a parallel trend observed globally; however, rates have been reported to be higher in North America ((18,19)). German hospitals have seen an approximate 50% decrease in CDI cases from 2015 to 2021 ((20)). Enhanced infection control practices, antimicrobial stewardship measures and improved surveillance and detection methods may have contributed to the overall decline seen in CDI rates ((19)). Additionally, CA-CDI patients with mild to moderate symptoms might not have any interactions with the healthcare system, resulting in underestimation of the true burden of CA-CDI ((19)).

In a representative sample of Canadian acute care hospitals, from 2018 to 2022, a 3.8% decrease in moxifloxacin resistance in both HA and CA-CDI populations is concordant with an overall decrease in the prevalence of RT027. Furthermore, moxifloxacin resistance remained lower (7.3% in 2022) than previously published weighted pooled resistance data for North America (44.0%) and Asia (33.0%) ((21,22)). The decline in RT027 prevalence from 2018 to 2022 may also have influenced the decline in CDI rates among CNISP hospitals as this ribotype has been associated with increased virulence and fluoroquinolone resistance ((23)). Additionally, the emergence of RT106 found worldwide and most predominantly in the US presents more fluoroquinolone resistance and higher recurrence rates. The potential emergence of resistant ribotypes warrants further surveillance, monitoring and investigation ((24,25)).

From 2018 to 2022, MRSA BSI rates decreased overall by 2.9% in the CNISP network. Although for three years, from 2019 to 2021, rates peaked at 1.13–1.16 infections per 10,000 patient days. Methicillin-resistant *Staphylococcus aureus* BSI is associated with increased morbidity and mortality, increased length of hospital stays and increased costs among admitted patients ((26–29)). The 16.4% decrease in clindamycin resistance among MRSA BSI isolates from 2018 to 2022 was likely associated with the decrease in the proportion of spa type t002 (CMRSA2 epidemic type) identified among tested isolates ((30)). The HA-MRSA BSI rates observed in the CNISP network from 2018 to 2022 (range: 0.42–0.50 infections per 10,000 patient days) were lower compared to those reported in Australian public hospitals from 2017 to 2020 (range: 0.71–0.76 infections per 10,000 patient days) ((31)).

The continued increase in the rate of patients hospitalized with CA-MRSA BSI observed in CNISP data from 2018 to 2022 suggests a growing CA-MRSA reservoir, both in Canada and globally ((32,33)). However, it is promising to see that over the last three years, from 2020 to 2022, CA-MRSA rates have been declining. Nonetheless, strategies to reduce or prevent MRSA infections in the community are still needed, especially in populations with increased risk of contracting CA-MRSA, such as children, athletes, incarcerated populations, seniors with comorbidities and people who inject drugs ((34,35)). Increasing injection drug use may indicate an emerging at-risk population for CA-MRSA, and as such, screening and eradication of the carriage of MRSA, may be effective in reducing the burden of MRSA BSI overall ((34–36)).

Vancomycin resistance related to VRE BSI has been shown to be a principal predictor of mortality and is associated with increased hospital burden ((37–39)). The VRE BSI rate observed in the CNISP network was highest in 2022 (0.36 infections per 10,000 patient days). The ST17 sequence type has contributed to the increased burden of VRE BSI in CNISP-participating hospitals by emerging as the predominant clone, overtaking ST1478. An increase in ST80 has also been seen in CNISP data, increasing from 11.7% in 2018 to 30.6% in 2022. The increase in ST80 seen in Canada is consistent with what has been observed in Sweden over the last three years, resulting in vanA-type and vanB-type outbreaks ((40)). The VRE BSI trends are further impacted by the number of high-risk patients admitted to hospital (e.g., bone marrow transplants, solid organ transplants, cancer patients, etc.) ((41)). Although there is a lack of recent data on VRE BSI rates in comparable jurisdictions, there have been increasing trends noted in Europe ((42–45)), which may be associated, in part, with the introduction and spread of new clones and gaps in infection prevention practices ((44–46)).

Carbapenemase-producing *Enterobacterales* infections are a significant threat to public health as they are becoming increasingly prevalent in healthcare environments worldwide ((47)). Active infection with CPE carries a high mortality rate, with the bacteria being resistant to many antibiotics, limiting treatment options for these patients ((5,48–52)). The Centers for Disease Control and Prevention and the World Health Organization have classified CPE as one of the most urgent antimicrobial-resistance threats ((52,53)). While the number of CPE infections increased from 2018 to 2022 in the CNISP network, incidence remained low ((54)). Data on the incidence of CPE infections in other countries, such as the United Kingdom, have also shown an increasing incidence of CPE infections ((54,55)). Similarly, the number of CPE isolates identified through laboratory surveillance associated with CPE infections has increased in Switzerland from 2013 to 2018 ((56)). More recently, a shift in the acquisition source of CPE has been observed within the CNISP network. Previously, CPE infections were mostly associated with international travel, but have recently become acquired domestically (85.3%) from 2020 to 2022. As a result, strict implementation of infection control measures, including screening in patients with a previous hospital admission domestically and abroad, are useful to reduce the transmission of CPE in Canadian acute care hospitals.

*Candida auris* is an emerging multi-drug resistant fungus that can cause HA invasive infections and outbreaks ((57)). It has been detected across multiple countries and continents including Canada, since its first detection in 2009 ((58–61)). *Candida auris* has been associated with outbreaks in healthcare settings in many countries, including Canada and the US, although outbreaks in Canada to date have been limited with few cases ((57)). Reported crude mortality for *C. auris* ranges widely from 15%–60% but is generally similar to other *Candida* species ((57–63)). Though still relatively rare in Canada, the US reported over 2,000 clinical cases and over 5,000 screening cases in 2022 ((64)). A survey examining *C. auris* preparedness within CNISP hospitals in 2018 found that most hospitals did not yet have laboratory protocols or infection prevention and control policies in place for detecting and controlling *C. auris* ((65)). The identification of *C. auris* in routine microbiology laboratories requires identification of *Candida* to the species level, which may not be routinely performed for isolates from non-sterile sites. Treatment options are limited for patients as one third of identified *C. auris* isolates in Canada were multidrug-resistant and additional resistance can develop during antifungal therapy ((66)). Therefore, rapid identification, screening for colonization in at-risk patients and strict implementation of infection prevention and control measures are required to reduce the transmission of *C. auris* in Canadian healthcare settings. Continued reporting on *C. auris* in Canada is important to assess and monitor the risk of this pathogen, in addition to identifying epidemiological and microbiological trends ((66)).

The COVID-19 pandemic has had a varied effect on the rates of HAIs in Canada and in the US ((67,68)). When looking at HAI rates before and during the COVID-19 pandemic, the data showed an immediate increase in HA rates of CDI while MRSA BSI, CPE infection and VRE BSI rates immediately decreased; however, COVID-19 pandemic status was not associated with lasting impacts on monthly rate trends in these infections ((69)). Pandemic-related improvements in hand hygiene, personal protective equipment practices, environmental cleaning, screening and infection control practices may have contributed to the decreases in rates observed over the reporting period ((70)).

## Strengths and limitations

The main strength of CNISP is the collection of standardized and detailed epidemiological and laboratory-linked data from 88 sentinel hospitals across Canada for the purpose of providing national HAI and AMR trends for benchmarking and to inform hospital infection prevention and control practices. It is important to note that data in this report include those from the early years of the COVID-19 pandemic; therefore, rates of HAIs and AMR in 2020 and 2021 may be impacted by changes in national, regional and municipal hospital-based infection prevention and control measures.

Epidemiological data collected by CNISP were limited to information available in patient charts. Hospital staff turnover may affect the consistent application of CNISP definitions when reviewing medical charts; however, these data were collected by experienced and trained infection prevention and control staff who receive periodic training with respect to CNISP methods and definitions. Furthermore, data quality assessments were conducted to maintain and improve data quality. These data may be subject to potential selection bias due to the exclusion of sites with missing or incomplete data throughout the study period. A limitation of *C. auris* surveillance is that detailed epidemiologic data are only available on patients identified at CNISP participating hospitals. From 2018 to 2022, CNISP coverage of Canadian acute care beds has increased from 32% to 35%, including increased representativeness in northern, community, rural and Indigenous populations.

## Next steps

Recruitment of rural and remote Canadian acute care hospitals to the CNISP network is an ongoing effort to improve the quality and representativeness of Canadian HAI surveillance data. Furthermore, the enhanced hospital screening practices survey is conducted annually to better understand and contextualize changes in HAI rates in the CNISP network. In recent years, CNISP has implemented surveillance for new and emerging pathogens, including *C. auris* and COVID-19. Studies are ongoing to assess the impact of the COVID-19 pandemic on HAI rates and AMR. The CNISP has recently made HAI and antibiotic-resistant organism rates publicly available in a dashboard format using Canada’s Health Infobase ((71)). Lastly, CNISP is also looking to study the feasibility of collecting data in the long-term care sector in Canada to examine status and scope of HAI/antibiotic-resistant organism surveillance, to better understand the burden of HAIs among this at-risk population. To further improve representativeness and generalizability of national HAI benchmark rates, CNISP has launched a simplified dataset accessible to all acute care hospitals across Canada to collect and visualize annual HAI rate data.

## Conclusion

Surveillance findings from a national sentinel network of Canadian acute care hospitals indicate that rates of MRSA BSI and CDI have decreased from 2018 to 2022, while rates of VRE BSI and CPE infections have increased. Few cases of *C. auris* were detected in Canada from 2012 to 2022. Consistent and standardized surveillance of epidemiologic and laboratory HAI data are essential to providing hospital practitioners with benchmark rates and informing infection prevention and control and antimicrobial stewardship policies to help reduce the burden of HAI and the impact of AMR in Canadian acute care hospitals.

## Appendix A Surveillance case definitions and eligibility criteria, 2022

### *Clostridioides difficile* infection

A “primary” episode of *Clostridioides difficile* infection (CDI) is defined either as the first episode of CDI ever experienced by the patient or a new episode of CDI that occurs greater than eight weeks after the diagnosis of a previous episode in the same patient.

A patient is identified as having CDI if:

· The patient has diarrhea or fever, abdominal pain and/or ileus AND a laboratory confirmation of a positive toxin assay or positive polymerase chain reaction (PCR) test for *C. difficile* (without reasonable evidence of another cause of diarrhea)

OR

· The patient has a diagnosis of pseudomembranes on sigmoidoscopy or colonoscopy (or after colectomy) or histological/pathological diagnosis of CDI

OR

· The patient is diagnosed with toxic megacolon (in adult patients only)

Diarrhea is defined as one of the following:

· More watery/unformed stools in a 36-hour period

OR

· More watery/unformed stools in a 24-hour period and this is new or unusual for the patient (in adult patients only)

Exclusion:

• Any patients younger than one year

• Any pediatric patients (aged one year to younger than 18 years) with alternate cause of diarrhea found (i.e., rotavirus, norovirus, enema or medication, etc.) are excluded even if *C. difficile* diagnostic test result is positive

*Clostridioides difficile* infection case classification:

Once a patient has been identified with CDI, the infection will be classified further based on the following criteria and the best clinical judgment of the healthcare and/or infection prevention and control practitioner.

Healthcare-associated (acquired in your facility) CDI case definition:

• Related to the current hospitalization:

o The patient’s CDI symptoms occur in your healthcare facility three or more days (or 72 hours or longer) after admission

• Related to a previous hospitalization:

o Inpatient: the patient’s CDI symptoms occur less than three days after the current admission (or fewer than 72 hours) AND the patient had been previously hospitalized at your healthcare facility and discharged within the previous four weeks

o Outpatient: the patient presents with CDI symptoms at your emergency room (ER) or outpatient location AND the patient had been previously hospitalized at your healthcare facility and discharged within the previous four weeks

· Related to a previous healthcare exposure at your facility:

o Inpatient: the patient’s CDI symptoms occur less than three days after the current admission (or fewer than 72 hours) AND the patient had a previous healthcare exposure at your facility within the previous four weeks

o Outpatient: the patient presents with CDI symptoms at your ER or outpatient location AND the patient had a previous healthcare exposure at your facility within the previous four weeks

Healthcare-associated (acquired in any other healthcare facility) CDI case definition:

· Related to a previous hospitalization at any other healthcare facility:

o Inpatient: the patient’s CDI symptoms occur less than three days after the current admission (or fewer than 72 hours) AND the patient is known to have been previously hospitalized at any other healthcare facility and discharged/transferred within the previous four weeks

o Outpatient: the patient presents with of CDI symptoms at your ER or outpatient location AND the patient is known to have been previously hospitalized at any other healthcare facility and discharged/transferred within the previous four weeks

· Related to a previous healthcare exposure at any other healthcare facility

o Inpatient: the patient’s CDI symptoms occur less than three days after the current admission (or fewer than 72 hours) AND the patient is known to have had a previous healthcare exposure at any other healthcare facility within the previous four weeks

o Outpatient: the patient presents with CDI symptoms at your ER or outpatient location AND the patient is known to have had a previous healthcare exposure at any other healthcare facility within the previous four weeks

Healthcare-associated CDI but unable to determine which facility:

The patient with CDI DOES meet both definitions of healthcare-associated (acquired in your facility) and healthcare-associated (acquired in any other healthcare facility) CDI, but unable to determine to which facility the case is primarily attributable to.

Community-associated CDI case definition:

· Inpatient: the patient’s CDI symptoms occur less than three days (or fewer than 72 hours) after admission, with no history of hospitalization or any other healthcare exposure within the previous 12 weeks

· Outpatient: the patient presents with CDI symptoms at your ER or outpatient location with no history of hospitalization or any other healthcare exposure within the previous 12 weeks

Indeterminate CDI case definition:

The patient with CDI does NOT meet any of the definitions listed above for healthcare-associated or community-associated CDI. The symptom onset was more than four weeks but fewer than 12 weeks after the patient was discharged from any healthcare facility or after the patient had any other healthcare exposure.

### Methicillin-resistant *Staphylococcus aureus* (MRSA) infection

MRSA bloodstream infection (BSI) case definition:

· Isolation of *Staphylococcus aureus* from blood

AND

· Patient must be admitted to the hospital

AND

· Is a “newly identified *S. aureus* infection” at a Canadian Nosocomial Infection Surveillance Program (CNISP) hospital at the time of hospital admission or identified during hospitalization

Infection inclusion criteria:

· Methicillin-susceptible *Staphylococcus aureus* (MSSA) or MRSA BSIs identified for the first time during this current hospital admission

· MSSA or MRSA BSIs that have already been identified at your site or another CNISP site but are **new** infections

Criteria to determine NEW MSSA or MRSA BSI:

· Once the patient has been identified with a MSSA or MRSA BSI, they will be classified as a new MSSA or MRSA if they meet the following criteria: more than 14 days since previously treated MSSA or MRSA BSI and, in the judgment of infection control physicians and practitioners, represents a new infection

Infection exclusion criteria:

· Emergency, clinic, or other outpatient cases who are **NOT admitted** to the hospital

Healthcare-associated (HA) case definition:

Healthcare-associated is defined as an inpatient who meets the following criteria and in accordance with the best clinical judgment of the healthcare and/or infection prevention and control practitioner:

· Patient is on or beyond calendar day 3 of their hospitalization (calendar day 1 is the day of hospital admission)

OR

· Has been hospitalized in your facility in the last 7 days or up to 90 days depending on the source of the infection

OR

· Has had a healthcare exposure at your facility that would have resulted in this bacteremia (using best clinical judgment)

OR

· Any patient who has a bacteremia not acquired at your facility that is thought to be associated with any other healthcare exposure (e.g., another acute-care facility, long-term care, rehabilitation facility, clinic or exposure to a medical device)

Healthcare-associated (HA) case definition (newborn):

· The newborn is on or beyond calendar day 3 of their hospitalization (calendar day 1 is the day of hospital admission)

· The mother was **NOT** known to have MRSA on admission and there is no epidemiological reason to suspect that the mother was colonized prior to admission, even if the newborn is fewer than 48 hours of age

· In the case of a newborn transferred from another institution, MSSA or MRSA BSI may be classified as HA your acute-care facility if the organism was **NOT** known to be present and there is no epidemiological reason to suspect that acquisition occurred prior to transfer

Community-associated case definition:

· No exposure to healthcare that would have resulted in this bacteremia (using best clinical judgment) and does not meet the criteria for a healthcare-associated BSI

### Vancomycin-resistant *Enterococcus* (VRE) infection

VRE BSI case definition:

· Isolation of *Enterococcus faecalis* or *faecium* from blood

AND

· Vancomycin minimum inhibitory concentration (MIC) of **at least** 8 µg/ml

AND

· Patient must be admitted to the hospital

AND

· Is a “newly” identified VRE BSI at a CNISP facility at the time of hospital admission or identified during hospitalization

**A newly identified VRE BSI** is defined as a positive VRE blood isolate more than 14 days after completion of therapy for a previous infection and felt to be unrelated to previous infection in accordance with best clinical judgment by infection control physicians and practitioners.

Exclusion criteria:

· Emergency, clinic, or other outpatient cases who are **not admitted** to the hospital

Healthcare-associated (HA) case definition:

Healthcare-associated is defined as an inpatient who meets the following criteria and in accordance with the best clinical judgment of the healthcare and/or infection prevention and control practitioner:

· Patient is on or beyond calendar day 3 of their hospitalization (calendar day 1 is the day of hospital admission)

OR

· Has been hospitalized in your facility in the last 7 days or up to 90 days depending on the source of the infection

OR

· Has had a healthcare exposure at your facility that would have resulted in this bacteremia (using best clinical judgment)

OR

· Any patient who has a bacteremia not acquired at your facility that is thought to be associated with any other healthcare exposure (e.g., another acute-care facility, long-term care, rehabilitation facility, clinic or exposure to a medical device)

### Carbapenemase-producing *Enterobacterales* (CPE) infection

Case eligibility:

· Patient is admitted to a CNISP hospital or presents to a CNISP hospital emergency department or a CNISP hospital-based outpatient clinic

· Laboratory confirmation of carbapenem resistance or carbapenemase production in *Enterobacterales* spp.

Following molecular testing, only isolates determined to be harbouring a carbapenemase are included in surveillance. If multiple isolates are submitted for the same patient in the same surveillance year, only the isolate from the most invasive site is included in epidemiological results (e.g., rates and outcome data). However, antimicrobial susceptibility testing results represent all CPE isolates (including clinical and screening isolates from inpatients and outpatients) submitted between 2018 and 2022; duplicates (i.e., isolates from the same patient where the organism and the carbapenemase were the same) were excluded.

### Candida auris

Patients admitted to a participating hospital or presenting to a hospital emergency department or a hospital-based outpatient clinic with laboratory confirmation of *C. auris* from any specimen.

Included in this surveillance project are all clinical or screening samples that were positive for *C. auris* by any method. Currently, *C. auris* can be identified by rRNA sequencing, Vitek MS MALDI-TOF (with either the clinical database v3.2 or later or the RUO database), or Bruker MALDI-TOF (with either the clinical database v6903 or later or the RUO database). The project also includes potential *C. auris* misidentifications or “No identification” as outlined in the **Table A1** below.

**Table A1:** Laboratory identification of *Candida auris*

Identification method	Identification of suspect isolates
Vitek MS MALDI Clinical database older than v3.2	*C. haemulonii* No ID/low discrimination *C. rugosa* (not a problem for v3.0 or later) *C. pulcherrima* (not a problem for v3.0 or later)
Bruker MALDI Clinical database older than v6903	No ID
Vitek 2 version 8.01	*C. haemulonii* *C. duobushaemulonii* No ID/low discrimination
Vitek 2 version before 8.01	*C. haemulonii* *C. duobushaemulonii* *C. lusitaniae* *C. famata* No ID/low discrimination
API 20C AUX	*Rhodotorula glutinis* (characteristic red colour not present) *C. sake* No ID/low discrimination
API Candida	*C. famata*
BD Phoenix yeast identification system	*C. haemulonii* *C. catenulata* No ID

Abbreviations: *C*., *Candida*; MALDI, Matrix-Assisted Laser Desorption Ionization; MS, mass spectrometry

## Appendix B

Supplemental figures and tables are available upon request to the author: cnisp-pcsin@phac-aspc.gc.ca

Figure S1: Number and proportion of patient admissions included in the Canadian Nosocomial Infection Surveillance Program by hospital type and size, 2022

Table S1.1: Cases and incidence rates of healthcare-associated and community-associated *Clostridioides difficile* infection by region, hospital type and hospital size, Canada, 2018–2022

Table S1.2: Antimicrobial resistance of healthcare-associated and community-associated *Clostridioides difficile* infection isolates, Canada, 2018–2022

Table S1.3: Number and proportion of common ribotypes of healthcare-associated and community-associated *Clostridioides difficile* infection cases, Canada, 2018–2022

Table S2.1: Cases and incidence rates of healthcare-associated and community-associated methicillin-resistant *Staphylococcus aureus* bloodstream infections by region, hospital type and hospital size, 2018–2022

Table S2.2: Antimicrobial resistance of healthcare-associated and community-associated methicillin-resistant *Staphylococcus aureus* bloodstream infection isolates, Canada, 2018–2022

Table S2.3: Number and proportion of select methicillin-resistant *Staphylococcus aureus* spa types (with corresponding epidemic types) identified

Table S3.1: Number of vancomycin-resistant *Enterococcus* bloodstream infections incidence rates by region, hospital type and hospital size, 2018–2022

Table S3.2: Number of healthcare-associated vancomycin-resistant *Enterococcus* bloodstream infections and incidence rates by region, hospital type and hospital size, 2018–2022

Table S3.3: Number and proportion of vancomycin-resistant *Enterococcus* bloodstream infections isolate types identified, 2018–2022

Table S3.4: Distribution of vancomycin-resistant *Enterococcus faecium* bloodstream sequence types, 2018–2022

Table S4.1: Number of carbapenemase-producing *Enterobacterales* infections and incidence rates by region, hospital type and hospital size, 2018–2022

Table S4.2: Number and proportion of main carbapenemase-producing pathogens identified

Table S4.3: Antimicrobial susceptibility testing for *Klebsiella pneumoniae* carbapenemase, 2019–2022

Table S4.4 Antimicrobial susceptibility testing for New Delhi metallo-β-lactamase, 2019–2022

Table S4.5: Antimicrobial susceptibility testing for OXA-48, Oxacillinase-48, 2019–2022
